# To cross or not to cross – thrushes at the German North Sea coast adapt flight and routing to wind conditions in autumn

**DOI:** 10.1186/s40462-019-0173-5

**Published:** 2019-10-31

**Authors:** Vera Brust, Bianca Michalik, Ommo Hüppop

**Affiliations:** Institute of Avian Research, An der Vogelwarte 21, 26386 Wilhelmshaven, Germany

**Keywords:** Thrushes, Songbirds, Migration, Stopover, Offshore, Routing

## Abstract

**Background:**

Although many aspects of passerine migration are genetically determined, routing appears to be flexibly adjusted to the conditions experienced on each individual journey. This holds especially true for routing decisions taken when confronted with large bodies of water. Once taken, these decisions can be hardly altered or revised. In this paper, we analysed stopover and routing decisions taken by three species of thrushes, blackbirds, redwings and song thrushes, at the German North Sea coast.

**Methods:**

Birds were equipped with radio-telemetry tags at stopover sites along the coast during autumn migration and subsequently tracked by an automated receiver network covering the coastline and islands of the German Bight.

**Results:**

The thrushes resumed migration in nights with a favourable northward wind component and clear skies. About 40% of the tagged individuals have taken an offshore instead of an alongshore oriented flight route. Routing decisions were influenced by the strength of the eastward wind component with offshore oriented flights taking place primarily under weak winds or winds blowing towards the west. Thrushes that took an offshore oriented route stopped over at the coast longer than those flying alongshore. Interestingly, offshore as well as alongshore oriented flights co-occurred within single nights and under comparable weather conditions.

**Conclusions:**

Migratory flight and routing decisions of thrushes at the German North Sea coast are highly dependent on weather, in particular wind. Still, we found evidence that weather may not be the sole reason for individual routes taken. Physical condition, morphology or animal personality lend themselves as possible additional factors of influence. Enabling a more detailed understanding of thrush migration over and along the German North Sea, our data help to better judge risks that migratory birds are facing when en route conditions are altered, for example by artificial obstacles such as offshore wind turbines.

**Electronic supplementary material:**

The online version of this article (10.1186/s40462-019-0173-5) contains supplementary material, which is available to authorized users.

## Background

Between individual variability in migratory routing has been increasingly documented over the last two decades (e.g [1, 2]), facilitated by the use of new tracking technologies that provide data in high spatial and temporal resolution [3]. Repeated tracking of single birds over multiple years additionally reveals high route variability within individuals, further demonstrating the existence of flexible en route adjustments of migratory tracks [4–8]. Recent evidence suggests that riskier journeys further increases between individual variability in routing adjustment [9–11].

During migration, many land birds cross the open sea, some species even over huge distances (overview in [12]). After departing for a flight over sea, they are compelled to fly non-stop until they reach land again. Consequently, crossing the open sea implies a higher risk than flights over land with regard to deteriorating weather conditions and exhaustion [13, 14]. Oversea flights should hence be taken with particular caution [15]. Nowadays, flights over the open sea may get even more dangerous with regard to the rapidly increasing number of offshore wind turbines and other artificial structures bearing the risk of collision (as reviewed in [12]). Radar studies suggested that nocturnally migrating birds usually do not alter their flight direction when encountering coastlines during the night [16, 17], except towards the end of the night and in the early morning, when they tend to re-orient towards the coast [18, 19]. This holds true especially for coasts deviating widely from the course of migration [20]. If coastlines are in closer accordance with the desired flight direction, detours along the coastlines are more likely taken [20, 21]. A partial rather than a full crossing of larger bodies of water often optimises flight routes with regard to energy and time expenditure [22].

The influence of weather on songbird migration is generally high, but gets even more important when attempting to cross a large body of water [15]. During unfavourable winds, a coastline can serve as a landmark, facilitating drift compensation [23–25]. Birds have been found to start flights over sea more likely under tailwind conditions [9, 26] and clear skies [27]. Besides wind, favoured weather conditions are characterised by low rates of precipitation and humidity as well as by low and sinking temperature, high and rising air pressure and good visibility (e.g. [21, 28, 29]). The combination of relevant factors, however, is generally less consistent and varies among species as well as over the season [30]. Especially in late autumn, weather factors other than wind become more important as the tendency to fly under less favourable conditions rises along with the urge to reach the wintering sites [31].

We investigated the flight behaviour of three nocturnally migrating songbird species, Eurasian blackbird (*Turdus merula*), redwing (*Turdus iliacus*) and song thrush (*Turdus philomelos*) caught at coastal stopover sites at the German North Sea coast. The expected direction of migration does not deviate too far from the course of the coastline in this area. Still, following the coastline comprises a detour as compared to a direct crossing of the open water. Although taking a non-stop flight over water across the German Bight lies well in the capability of our studied species, it is generally known that detours are often taken to reduce risky passages [22]. During the night, the amount of travelling birds in the German Bight area was estimated higher at coastal islands as compared to the offshore island of Helgoland [32]. Thrushes migrating along or across the German Bight during autumn are foremost Fennoscandian breeders on their way to wintering grounds in western or south western Europe [33–35]. All three species investigated in this study are known to migrate across the open water of the German Bight in large numbers as indicated for example by bird ringing activities on the island of Helgoland [36] or by call recordings at an offshore-research platform in the North Sea [37].

While the overall direction of thrush migration during autumn is well known in this region [38, 39], data on individual stopover and routing are lacking. This gap results from methodological deficiencies in the available studies, which either lack the opportunity to follow individual flight tracks (visual and acoustic observations, ringing, collision victim counts), or lack information on the individual during tracking (radar). Here, we use an extended radio-telemetry receiver network [40] covering the German Bight coastline and several islands. Combining radio-tracking data with meteorological data, we are able to shed light on the conditions underlying individual departure and routing decisions of thrushes in this area for the first time. We expect the onset of migratory flights at the coast to strongly depend on weather conditions. Flight routes should accordingly be adjusted to weather, i.e. flights out to sea should occur under supportive wind conditions and good visibility.

## Methods

**Fig. 1:**
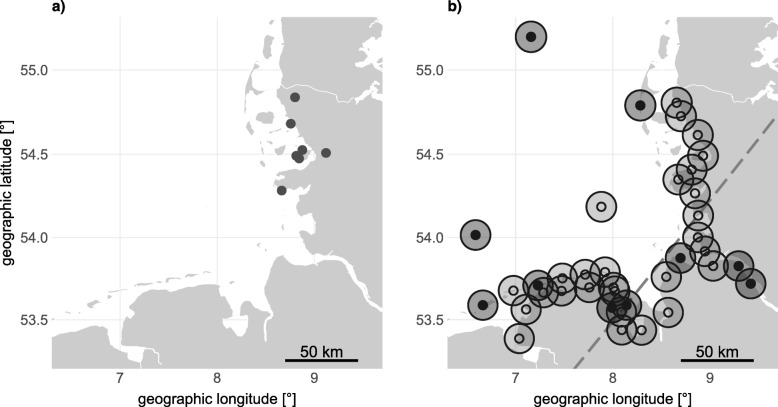
**a** Tag deployment sites of thrushes at the coast of Schleswig-Holstein, Germany, during autumn migration 2017 and 2018. **b** Locations of automated radio-telemetry receivers at the German North Sea coast (open circles: receiver stations active in 2017 and 2018, black dots: additional receiver stations active in 2018). Semi-transparent circles are presumed 10 km detection radii of antennas. Dashed grey lines indicate threshold latitude and longitude for flight categorisation

We caught 152 individuals from the three species of thrushes with mist nets during autumn migration from Sept 28th to Oct 24th 2017 and from Oct 3rd to Oct 17th 2018 (Table 1a). Catching took place at seven different spots scattered along the German North Sea coast in Schleswig-Holstein (Fig. 1a). Spots reflected stopover sites close to the coast, chosen in order to catch the birds on their migratory path prior to reaching the open water. Birds were equipped with individually coded radio-telemetry transmitters of type NTQB (Lotek Wireless Inc., Newmarket, ON, Canada) directly after catching. Leg loop harnesses consisted of non UV resistant elastic rubber band and were expected to fall off the bird after a few weeks as the rubber soon gets brittle [41]. Tags including harnesses weighted about 0.29 g. Movements were subsequently tracked by an array of automated radio-telemetry receivers covering the German North Sea coastline and islands located in the German Bight (Fig. 1b). Our receivers are part of the Motus Wildlife Tracking System, a worldwide collaborative radio-telemetry network [42]. For the extent of the full receiver network and for detailed information on each receiver, please visit motus.org.

**Table 1:** Number of thrushes tagged from Sept 28th to Oct 24th 2017, and from Oct 3rd to Oct 17th 2018, respectively (a) and subsequently detected flights that took place from Oct 18th to Nov 13th 2017 and from Oct 17th to Nov 17th 2018 (b) per species and year

a)	tagged birds				
	species	2017	2018	total	detected
	blackbird	49	0	49	19
	redwing	14	34	48	37
	song thrush	33	22	55	44
b)	flights				
	species	2017	2018	alongshore	offshore
	blackbird	5	0	4	1
	redwing	2	15	10	7
	song thrush	13	14	16	11

### Data analyses

All data analyses were performed using R 3.5.2 [43]. ESRI shape files of the shorelines were downloaded from the GSHHG database of the NOAA National Centers for Environmental Information [44]. Weather data were obtained from the Reanalysis I datasets of the US National Centers for Environmental Prediction (NCEP) via the RNCEP package [45].

Individual detection data were automatically extracted from receiver recordings by motus.org [42] and were subsequently downloaded via the Motus R package [46]. Detailed descriptions of all parameters provided by motus.org for each extracted detection can be found in ([47], appendix A). Prior to all subsequent analyses, our detection data, comprising 329 NTQB tags used in all of our projects prior to January 2019, were checked for false positives. Based on basic data filtering provided by motus [47], we developed a filtering method to best fit the specifications of our dataset by including each run’s mean burst slop, mean frequency standard deviation, mean slop, number of runs recorded per receiver-hour-bin, proportion of short run lengths (i.e. < 4) per receiver-hour-bin, proportion of recordings with short run lengths per tag ID, tag model, number of continuous runs recorded in ±25 min of a run’s mean timestamp as well as the corresponding number of antennas. We used two separate routines for data recorded by the German Bight receiver network and by the Netherlands’ receiver network, respectively, as receiver properties differ between the two networks. Based on these analyses, we used the filtered subset of all detections of thrushes during autumn 2017 and 2018 including all data with a predicted probability estimate of being a false positive detection below 0.8 (please see Additional file 2 for full details on the data filtering process)

We subsequently identified continuous movements, from now on referred to as “flights”. A flight was classified as covering a distance of at least 35 km or as being recorded by a minimum of three different receivers with consecutive detections in less than 7 hours. We restricted our analyses to flights starting in the area of the German Bight, resulting in 53 identified flights of 49 individual birds. As we were interested in initial routing decisions of different individuals, we included only the first flight of each bird into subsequent analyses. Flights were classified as either alongshore or offshore oriented. An offshore oriented flight started at geographic latitudes above 54.135° N and ended at geographic longitudes below 8.08° E or included detections by receivers on the island of Helgoland, located approximately 50 km off the mainland, or by receivers on the offshore research platforms FINO 1 and FINO 3 (Fig. 1b). All other flights were classified as alongshore. This classification is regarded to be rather conservative by ignoring possible offshore flights over shorter distances or in closer proximity to the coast.

#### Onset of flights

To reveal conditions that might influence an individual to embark on a flight, we applied a Cox Proportional Hazards model (CoxPH, [48, 49]). In order to analyse the effect of weather on the onset of flights, the flights dataset was expanded to include weather data for each day and location of each bird on that day from tag deployment onwards until flight (864 data points). We included the following weather parameters into the first model (Additional file 1: Table S1): eastward (u) and northward (v) wind vectors, relative humidity, air pressure and air temperature, precipitation rate and total cloud cover. Data were linearly interpolated by NCEP in relation to surface or pressure levels of 1000 hPa to the time of sunset and to the geographic coordinates of the flight start point. Precipitation rate and total cloud cover are provided by NCEP as six hour averages. We used the six hour averages around local sunset, i.e. between 12 and 18 h UTC. Prior to analysis, these parameters were checked for non-collinearity by their variance inflation factor (vif, R package usdm, [50]). Additional parameters included into the model were species, Julian day of year, and year. All numeric variables included were z transformed on species level. Since weather parameters are highly time-dependent, the CoxPH model was performed using its (start, stop] form with defining time steps in single days, and with defining flight as event (R package survival, [49]). The data were clustered per individual. Results of the initial model fit can be found in Additional file 1: Table S1. The most parsimonious model was identified according to AIC (R package MuMIn, [51]). For the resulting Cox regression fit of the first weather model, the proportional hazards assumption was tested, which assumes a constant effect of the calculated coefficient over time. The species’ coefficients correlated, however, significantly with time, violating this assumption and necessitating a subdivision. By graphical investigation, we identified three distinct periods of time (Additional file 3: Figure S2). The original data set was cut accordingly at 10 and 21 days. The stratified time-dependent coefficient was included in the final model in order to meet the model’s assumption of proportional hazards as described by Therneau [49]. Flight probabilities were defined as 1 - ‘survival’ probability and were predicted for each parameter of interest by setting the other parameters to their mean values.

#### Flight routes

In order to identify factors influencing a bird to fly alongshore or to cross the open waterbody, we started with setting up a binomial GLM: The initial model included presumably relevant weather parameters interpolated for the geographic coordinates of the flight start, i.e. u- and v-wind components, relative humidity, change in air pressure as well as change in air temperature over the last 24 h, and total cloud cover as well as the minimum stopover duration, i.e. time difference in days between the day of tag deployment and day of flight, and onset of flight in relation to sunset (see Additional file 1: Table S2). Data were z transformed on species level and year and species were included as additional fixed factors. A set of models with combinations of fixed effects was created and the most parsimonious model according to AIC was identified. Nagelkerke’s pseudo R^2^ ([52] for a more recent discussion see also, [53]) was calculated using the lrm function of the R package rms [54].

## Results

### Onset of flights

For 49 individuals at least one continuous flight could be recorded by the automated receiver network (Table 1). The thrushes resumed migration after two to 34 days of stopover post tag deployment. On average, they stayed for 16 ± 7.5 days (mean ± sd) at the coast of Schleswig-Holstein. Birds embarked on flights at only 22 different nights throughout the two autumn seasons. On 40% of these nights (13/22) only one bird resumed migration. The other nights were chosen by at least two and up to a maximum of nine birds.

**Fig. 2:**
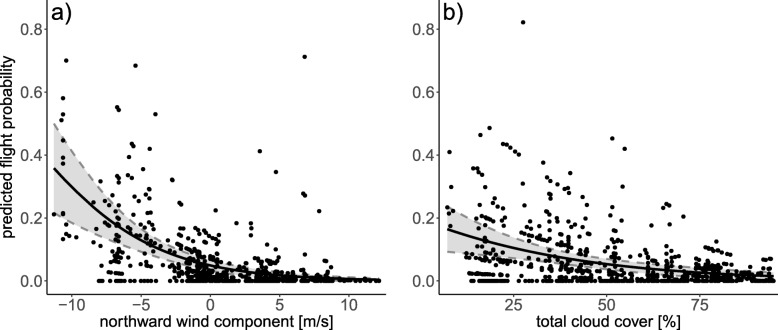
Predicted flight probability of thrushes during autumn migration at the German Bight in relation to **a**) prevailing northward (v) wind conditions in m/s, and **b**) prevailing total cloud cover in %. Flight probabilities (black dots) relative to the respective weather parameter were predicted by setting the values of the other parameters to their mean. Solid lines represent fitted regression lines with 95% confidence intervals (grey shaded area)

**Fig. 3:**
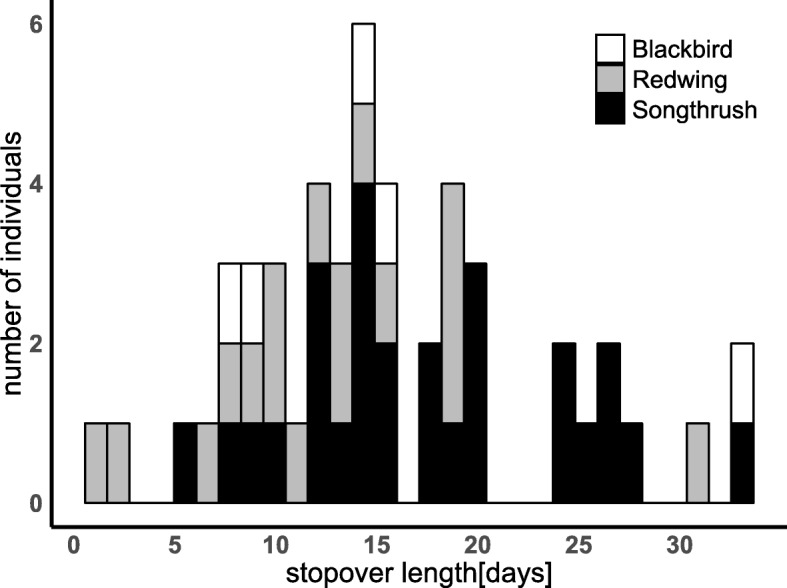
Histogram of the stopover length of the three species of thrushes

Individual departure decisions were significantly influenced by the northward wind component and total cloud cover. Full results of the initial CoxPH model fit can be found in Additional file 1: Table S1 of the supplement, final model results in Table 2. Thrushes flew more likely under weak and southward winds (Fig. 2a) and less clouded skies Fig. 2b). Additionally, stopover length differed between the three species (Table 2, Fig. 3). During the first stopover period as defined from the CoxPH model, mean minimum stopover durations (up to 9 days) did not differ in length between species. From birds that stayed between 10 and 20 days, redwings departed quicker than song thrushes. One blackbird stayed longer than 20 days and stayed even longer than the seven song thrushes that departed after more than 20 days of stopover. Notably and although precipitation did not significantly affect departure in the CoxPH model, the precipitation rate measured on flight days never exceeded 0.52 mm/h with most of the birds (48/49) flying at precipitation rates below 0.17 mm/h and 45% (22/49) even at times with no precipitation at all. Air pressure ranged between 986 and 1034 hPa during the two autumn seasons. Most birds (96%; 47/49) flew at air pressures above 1005 hPa. Air temperatures ranged between 3.2 and 20.3 °C. 65% (32/49) of the birds flew at temperatures above 8 °C. Relative humidity ranged between 57.9 and 97.2% with 65% of the birds (32/49) flying at a relative humidity below 80%.

**Table 2:** Results of the most parsimonious CoxPH model investigating the influence of weather parameters on individual departure decisions after model selection according to AIC and stratifying the species’ effect into three distinct time periods

parameter	β	exp(β)	se(β)	z	p
air pressure	0.24	1.27	0.17	1.39	0.164
total cloud cover	−0.54	0.58	0.21	−2,62	0.009 **
northward (v)-wind component	−1.03	0.36	0.20	−5.27	< 0.001 ***
time period 1: species level ‘blackbird’ (compared to species level ‘song thrush’)	1.65	5.23	0.93	1.77	0.076 •
time period 2: species level ‘blackbird’ (compared to species level ‘song thrush’)	−0.70	0.49	0.79	−0.90	0.371
time period 3: species level ‘blackbird’ (compared to species level ‘song thrush’)	−2.72	0.07	1.22	−2.24	0.025 *
time period 1: species level ‘redwing’ (compared to species level ‘song thrush’)	1.42	4.15	0.74	1.93	0.054 •
time period 2: species level ‘redwing’ (compared to species level ‘song thrush’)	0.85	2.35	0.41	2.07	0.039 *
time period 3: species level ‘redwing’ (compared to species level ‘song thrush’)	−0.27	0.77	1.70	−0.15	0.039 *
time period 1: species level ‘blackbird’ (^a^compared to species level ‘redwing’)	0.23	1.26	0.86	0.27	0.788
time period 2: species level ‘blackbird’ (^a^compared to species level ‘redwing’)	−1.56	0.21	0.85	−1.84	0.065 •
time period 3: species level ‘blackbird’ (^a^compared to species level ‘redwing’)	−2.45	0.09	1.96	−1.26	0.210

• *p* < 0.1, * p < 0.05, **p < 0.01, *** *p* < 0.001

*R*^*2*^ *= 0.073 (max possible: R*^*2*^ *= 0.299), AIC = 245.404*

^*a*^ *note that comparison between species levels ‘blackbird’ and ‘redwing’ has been achieved by re-calculating the model with manually re-ordered factor levels*

### Flight routes

**Fig. 4:**
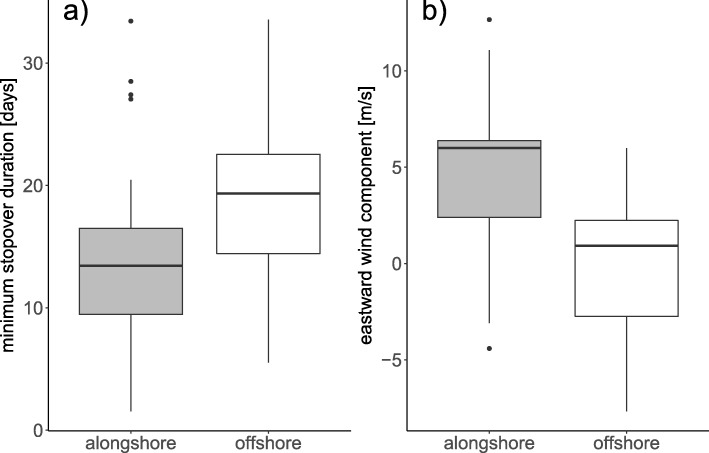
Routing decisions of thrushes at the German Bight were significantly influenced by **a**) minimum stopover duration in days, and **b**) eastward (u) wind component in m/s, Tukey style box and whisker plot

From the 49 flights included in the analysis, 30 were oriented alongshore and 19 crossed the open water (Table 1b). Differences in routing occurred between and also within flight nights. In six out of the nine nights with at least two birds flying, individuals took different routes. In a specific case when six individuals, four song thrushes and two blackbirds, started their flight within the range of the same receiver station in a single night and within a period of 3.17 h, five birds followed the coastline while one song thrush flew offshore. Out of the nine parameters included in the initial model (Additional file 1: Table S2), the minimum stopover duration and eastward wind component remained in the final model and significantly influenced routing decision (Table 3). In more detail, offshore flights occurred more likely after longer stopovers at the coast (Fig. 4a). Weak or westward oriented winds favoured offshore oriented flights, whereas eastward oriented winds favoured flights alongshore (Fig. 4b).

**Table 3:** Result of the final binomial GLM on taken routes after automated model selection of the initial models according to AIC

parameter	estimate	se	z	p
intercept	−0.57	0.36	−1.62	0.106
minimum stopover duration	0.90	0.45	2.01	0.045 *
eastward (u)-wind component	−1.21	0.43	−2.83	0.005 **

*p < 0.05, **p < 0.01, *** *p* < 0.001

*pseudo R*^*2*^ *= 0.383, AIC = 55.17*

## Discussion

Thrushes at coastal stopover sites at the German Bight waited for favourable wind conditions and clear skies in order to resume migration. Once in the air, solely the eastward wind component was identified to influence the orientation of migration either offshore or along the coast. Flights across the open sea took place in about 40% of the cases, predominantly under weak winds or winds blowing offshore. Onshore oriented winds, in contrast, favoured flights along the coastline. Interestingly, birds that stayed longer at the stopover sites were more likely taking the offshore oriented route as compared to individuals that continued migration more quickly.

### Onset of flights

Winds in Central Europe are mostly unfavourable during autumn migration as the prevailing west and southwest wind direction [55, 56] is experienced as head or crosswind by birds migrating south-westwards. Nights with favourable northeasterly wind conditions are rather exceptional in this region. We found a close link between departure decisions and the northward wind component, indicating that thrushes preferred weak and supportive winds to resume migration from the coast. This finding goes well in line with a generally higher intensity of thrush migration in autumn observed under tailwind conditions by radar at the Swedish coast [57], call recordings offshore [37] and catching numbers at the island of Helgoland [58]. An additional hint for the need to wait for favourable winds in our study area is that, although tagged at different locations and days throughout the season, several thrushes chose similar nights to resume migration. Autumn thrush migration has been found to be additionally favoured by clear skies along with low air temperatures [57], decreasing [57] or low humidity [37] and scarce rainfall [58]. While our data confirm, besides northerly winds, a preference for clear skies, an influence of the other parameters was not found. The influence of weather parameters other than wind has been considered before as minor when directly compared to wind [59]. The detection of these effects may thus be impossible in our sample of individually radio-tracked birds as compared to the huge sets of available radar, visual, acoustic and catching data used in the studies mentioned above. Particularly precipitation, cloud cover and humidity can be fairly locally distinct [60], and thus their effects on bird migration may be underestimated when working with interpolated weather data. Also, these factors are less reliably predicted by the NCEP Reanalysis model [61]. Still, it is interesting to note that although the overall precipitation rate was relatively low during the whole study period, precipitation was even lower in flight nights, indicating a selection for “good” weather.

Birds resting at stopover sites at the coast are accordingly likely to prolong their stay until favourable conditions occur. In line with the predomination of unfavourable winds in our study area, we observed in our thrushes an average minimum stopover duration of 16 days from tag deployment. Passerine stopovers in general are usually within the range of one to 15 days [62]. A mark-recapture analysis at the Baltic Sea coast estimated roughly 6 days as the mean minimum stopover of song thrushes and redwings in autumn [63]. If birds are recaptured for stopover estimation, their stays are most likely well underrated [62]. As the birds in our study were automatically detected and minor landscape movements were also included, our measurements of stopover duration in the three species is likely to be more precise. Besides having to wait for favourable conditions, a stopover period of up to 34 days as measured in our study may reflect a slower and likely less energetically costly migration, which is believed to be favoured by many species in autumn (e.g. [64–67]). Differences in stopover duration actually appear to be the main biological mechanism behind differences in overall migration speed [67].

### Flight routes

About 40% of flights of thrushes were categorised as offshore oriented. Our network of receivers however is predominantly coastal. It is consequently more likely to detect birds at the coast following the coastline as compared to birds heading offshore. Additionally, our definition of offshore flights is rather conservative. The estimated proportion of offshore oriented flights should consequently be considered with caution. Given the geographic conditions at our study site, resuming migration by crossing the open water body of the German Bight can save some but not a lot of time and energy but is riskier than following the coastline. Consequently, the tagged thrushes may likely neglect this shortcut, especially under non-ideal conditions. A greater advantage of crossing can be achieved if a bird at our tag deployment sites directly crossed to the Dutch coast. We detected only a few birds that took this decision, possibly due to the less dense network of receivers in the Netherlands at the time of study (www.motus.org). Once a thrush had departed at the coast, its routing was highly associated with wind conditions. While most birds preferred to embark on flights under southward oriented winds, flights over the open water occurred predominantly under weak winds or winds with a westerly component, i.e. blowing offshore. Shamoun-Baranes and van Gasteren [68] found rare mass migration events over the North Sea to solely take place under supportive wind conditions. A preponderant role of wind direction in routing decisions of thrushes is thus well comprehensible.

As the birds already selected favourable conditions to take-off, it is not surprising that the routing decision itself did not depend on any weather parameter other than the eastward wind component. The occurrence of both, along- and offshore oriented flights within single nights may still result from differences in weather conditions between take-off sites or from changing conditions, e.g. turning winds over the course of the night. However, given that also thrushes resuming migration from the same place and in close timely proximity took different routes, an interplay between weather and individual factors [69], like for example fuel load at take-off [70, 71], state of health [72] or personality [73] is most likely causing each individual’s routing decision. Our findings favour the assumption of routings to be a particularly flexible and complex part of migration that is less genetically determined than, for example, the timing of migration [6].

The time of flight onset during the night did not influence the thrushes’ routing. Bruderer and Liechti [18], who observed autumn migration at the Mediterranean coast by radar, found birds to predominantly fly offshore in the early night hours, whereas later individuals more often refrained from taking this route. While the authors most likely observed a majority of birds amidst their migratory flight, the birds in our study obviously terminated their flight when encountering the coastline in the first instance. Consequently, they were able to time the beginning of their next flight as desired. Offshore migration of thrushes in autumn takes place throughout the whole night, peaks at midnight and rarely continues into daylight hours [37, 74]. Interestingly, offshore oriented thrushes did not set flights on earlier, indicating that the findings of Bruderer and Liechti [18] were most likely caused by birds that had already undertaken a longer journey before they refrained from flying offshore in the early morning hours. The thrushes that took an offshore oriented route in our study resumed stopover longer than individuals flying alongshore. This may indicate that the birds were aware of the open water body they were facing and decided for a specific route already well ahead of the actual onset of their flight.

## Conclusion

Migratory flight and routings of thrushes at the German North Sea coast are highly dependent on weather conditions, primarily on wind. Still, they do hold an additional individual component factored into each birds’ decision. Following the individual tracks of thrushes enabled us to comprehend the birds’ routing and to estimate for the first time actual proportions of birds taking a specific route. Prior studies on bird migration over the German Bight were already able to estimate local numbers of individuals flying offshore from count and radar data [75–77]. Their results give an impression of the numbers of birds in general and more particularly of passerines crossing the open water of the German Bight. Our data add another piece to the puzzle of understanding passerine offshore migration. The more detailed our understanding of migration at the German Bight gets, the better we will be able to judge the risks migrating birds are facing when en route conditions are altered, for example by artificial obstacles such as offshore wind turbines.

## Additional files

Additional file 1: **Table S1** Results of the initial CoxPH model investigating the influence of weather parameters on individual onsets of flights. **Table S2** Result of the initial binomial GLM investigating the effect weather and stopover parameters on individual flight routes. (DOCX 17 kb)

Additional file 2: Data cleaning - Identifying false positive detections. (DOCX 62 kb)

Additional file 3: **Figure S2**. Graphical test of the proportional hazards assumption of the β coefficients calculated in the Cox Proportional Hazards model, which showed a significant correlation over time. The graph displays Schoenfeld residuals (black dots) for β of species ‘redwing’ (a) and ‘song thrush’ (b) over time along with a smooth regression line and its 95% confidence intervals (grey shaded area). Zero as well as β calculated from the model are indicated as horizontal dot-dashed and dashed lines, respectively. Vertical lines indicate the post-hoc cut of the data at 10 and 21 days, respectively. (PDF 8 kb)

## Data Availability

The dataset used during the current study is available from the corresponding author on reasonable request.
