# Supplementary material for: To cross or not to cross – thrushes at the German North Sea coast adapt flight and routing to wind conditions in autumn
Source: Mov Ecol. 2019 Oct 31;7:32. doi: 10.1186/s40462-019-0173-5 (PMC6824093; doi:10.1186/s40462-019-0173-5)
Supplement: Supplementary file 1 — Table S1 Results of the initial CoxPH model investigating the influence of weather parameters on individual onsets of flights. Table S2 Result of the initial binomial GLM investigating the effect weather and stopover parameters on individual flight routes. (DOCX 17 kb) [file 40462_2019_173_MOESM1_ESM.docx]

SUPPLEMENT

Table S1: Results of the initial Cox PH model investigating the influence of weather parameters on individual departure decisions.

| parameter | β | exp(β) | se(β) | z | p |
| --- | --- | --- | --- | --- | --- |
| day of year | 0.361 | 1.435 | 0.358 | 1.355 | 0.175 |
| eastward (u) wind component | -0.233 | 0.792 | 0.198 | -1.491 | 0.136 |
| northward (v) wind component | -0.991 | 0.371 | 0.278 | -3.634 | < 0.001*** |
| air pressure | 0.461 | 1.586 | 0.225 | 2.411 | 0.016* |
| precipitation rate | 0.282 | 1.326 | 0.284 | 1.144 | 0.252 |
| total cloud cover | -0.412 | 0.662 | 0.231 | -2.059 | 0.039* |
| air temperature | -0.007 | 0.993 | 0.341 | -0.022 | 0.982 |
| relative humidity | -0.012 | 0.988 | 0.256 | -0.046 | 0.963 |
| year (level '2018') | 0.205 | 1.228 | 0.453 | 0.565 | 0.572 |
| species (level 'Redwing') | 1.194 | 3.301 | 0.767 | 1.484 | 0.138 |
| species (level 'Song Thrush') | 0.475 | 1.608 | 0.630 | 0.734 | 0.463 |

R² = 0.069 (max possible R² = 0.299), AIC = 253.079

Table S2: Result of the initial binomial GLM investigating the effect of weather parameters on individual routing.

| parameter | estimate | se | z | p |
| --- | --- | --- | --- | --- |
| intercept | -1.72 | 1.23 | -1.40 | 0.163 |
| eastward (u) wind component | -1.70 | 0.79 | -2.13 | 0.033 * |
| northward (v) wind component | -0.05 | 0.70 | -0.07 | 0.948 |
| relative humidity | -0.76 | 0.80 | -0.95 | 0.343 |
| 24 hrs change in air pressure | -0.71 | 0.72 | -0.99 | 0.325 |
| 24 hrs change in air temperature | 0.28 | 1.12 | 0.25 | 0.806 |
| total cloud cover | -0.60 | 0.67 | -0.90 | 0.370 |
| year (level ‘2018’) | -1.07 | 1.48 | -0.73 | 0.469 |
| minimum stopover duration | 0.94 | 0.70 | 1.35 | 0.178 |
| take-off time in relation to sunset | -0.62 | 0.59 | -1.06 | 0.289 |
| species (level ‘redwing’) | 1.76 | 1.79 | 0.98 | 0.325 |
| species (level ‘song thrush’) | 1.58 | 1.49 | 1.06 | 0.288 |

pseudo R² = 0.484, AIC = 67.847
