# Supplementary material for: To cross or not to cross – thrushes at the German North Sea coast adapt flight and routing to wind conditions in autumn
Source: Mov Ecol. 2019 Oct 31;7:32. doi: 10.1186/s40462-019-0173-5 (PMC6824093; doi:10.1186/s40462-019-0173-5)
Supplement: Supplementary file 2 — Data cleaning - Identifying false positive detections. (DOCX 62 kb) [file 40462_2019_173_MOESM2_ESM.docx]

Supplement B: Data cleaning - Identifying false positive detections

False positive detections can arise from multiple sources of electromagnetic noise. They are liberally allowed during the process of automated tag filtering at Motus so as to minimise the risk of missing any true tag detections (47, Chapter 5). In order to sort out false positive detections from these pre-filtered data, we predicted the probability of any detection to be a false positive: We started with a merged dataset of detection data from all our projects, comprising signals of 329 individual NTQB tags. In a first step, each detection was classified a priori as either ‘good’ or ‘bad’ (Phil Taylor, pers. comm.; 47, Chapter 5). In a second step, we identified possible diagnostic physical parameters provided by Motus (please see 47 Appendix A for a detailed description of the single variables) to characterise the quality of a detection. With these parameters, we thirdly ran a logistic regression (binomial glm) in order to predict the overall probability of being a ‘bad’ detection. Finally, we used the probability estimates provided from the model to define a threshold for data filtering. All three steps are explained in full detail below.

Step 1 – A priori classification of a subset of detections

We performed our classification on 'runs', which are series of consecutive presumed bursts (coded signals) of a given tag detected by the same antenna at the same receiver (47, Appendix A). Three typical features of false positive detections were used for a priori classification: a short run length, a high percentage of missed pulses within a run and a large deviation of the recorded mean burst interval from the tag's burst interval (see 47 Appendix A for detailed description of run length and burst interval). We conservatively classified ‘good’ runs to miss less than 25% of the pulses or have deviations in the mean burst intervals that are smaller than the tag burst intervals themselves. ‘Bad’ runs had a run length smaller than four or more than 75% of the pulses are missing or their recorded mean burst intervals exceeded the tag burst interval more than 3 times. All other runs were classified as 'unassigned'.

Step 2 – Identification of possible diagnostic parameters for all detections

To better describe the quality of each recorded run, we calculated mean values for additional parameters provided for each detection by Motus: ‘burstSlop’, ‘freqsd’, and ‘slop’ (see 47 Appendix A for detailed explanations of these variables). Additionally, we calculated some more parameters of potential interest. Noisiness for each corresponding receiver was included as ‘runs per receiver hour’, ‘proportion of short run lengths per runs per receiver hour’, and ‘tag's proportion of short run lengths’. Continuity of the recording was reflected as the 'number of consecutive runs' assigned to the same tag and the 'number of recording antenna ports' within a time frame of plus or minus 25 min around the mean time stamp of the given run. Furthermore, as tag properties varied between seasons, we used the tag model type as an additional diagnostic parameter.

Step 3 – Prediction of the probability of being a false positive

We transformed the a priori category of our data into a binary variable by using only the ‘bad’ and ‘good’ detections from step 1 in order to predict the probability of being a ‘bad’ detection. We ran two binomial glms including the variables described in steps 1 and 2 for the detections by the German receiver network and the Netherlands' receiver network separately, as the receiver properties differ between the two networks. Tables S1 and S2 show the respective model outputs.

Table S1: Outcome of the logistic regression for detections (‘runs’) of NTQB tags by the German receiver network.

| variable | estimate | std. error | z | p |  |
| --- | --- | --- | --- | --- | --- |
| intercept | -4.1731 | 0.1929 | -21.6281 | < 0.0001 | *** |
| mean burstSlop (s) | 14.6368 | 8.2835 | 1.7670 | 0.0772 |  |
| mean freqsd (kHz) | 4.5211 | 1.9866 | 2.2758 | 0.0229 | * |
| mean slop (ms) | 2405.8085 | 146.2584 | 16.4490 | < 0.0001 | *** |
| n continuous runs | 0.0005 | 0.0001 | 4.6251 | < 0.0001 | *** |
| n ports | 0.0111 | 0.0468 | 0.2377 | 0.8121 |  |
| tag's proportion of short runs | 0.5762 | 0.1532 | 3.7606 | 0.0002 | *** |
| runs per receiver hour | -0.0195 | 0.0013 | -15.4262 | < 0.0001 | *** |
| proportion of short runs | 6.8022 | 0.1486 | 45.7603 | < 0.0001 | *** |
| tag model NTQB-2 | 0.3044 | 0.1355 | 2.2471 | 0.0246 | * |
| tag model NTQB2-1 | 0.3991 | 0.1271 | 3.1412 | 0.0017 | ** |
| tag model NTQB2-2 | 0.3714 | 0.1339 | 2.7738 | 0.0055 | ** |

AIC = 7668.5

Table S2: Outcome of the logistic regression for detections (‘runs’) of NTQB tags by the Netherlands' receiver network.

| variable | estimate | std. error | z | p |  |
| --- | --- | --- | --- | --- | --- |
| intercept | 2.7072 | 990.2879 | 0.0027 | 0.9978 |  |
| mean burstSlop (s) | 26.9616 | 37.8359 | 0.7126 | 0.4761 |  |
| mean freqsd (kHz) | 16.6468 | 7.1207 | 2.3378 | 0.0194 | * |
| mean slop (ms) | 1554.7625 | 428.4784 | 3.6286 | 0.0003 | *** |
| n continuous runs | 0.0002 | 0.0002 | 0.9705 | 0.3318 |  |
| n ports | 0.3332 | 0.1743 | 1.9121 | 0.0559 |  |
| tag's proportion of short runs | 0.3765 | 1.4760 | 0.2551 | 0.7987 |  |
| runs per receiver hour | -0.0216 | 0.0057 | -3.7835 | 0.0002 | *** |
| proportion of short runs | 5.7869 | 0.9197 | 6.2923 | < 0.0001 | *** |
| tag model NTQB-2 | -7.7736 | 990.2879 | -0.0078 | 0.9937 |  |
| tag model NTQB2-1 | -8.3086 | 990.2877 | -0.0084 | 0.9933 |  |
| tag model NTQB2-2 | -7.9096 | 990.2876 | -0.0080 | 0.9936 |  |

AIC = 222.86

We then used the model outcome to predict the probability estimates for the whole dataset. We plotted the probability for each of the categories defined in step 1 to visually check the validity of our predictions (Fig. S1) and to define a threshold value to filter out false positives from our data. Based on these analyses, we used the filtered subset of all detections of thrushes during autumn 2017 and 2018 including all data with a predicted probability estimate of being a false positive detection below 0.8. The full data set analysed for this paper comprised 126255 detections of which 31374 were filtered out as false positives by using this threshold.

Fig. S1: Densities of estimated probabilities of ‘good’ and ‘bad’ detections as predicted by the logistic regression from all detection data of NTQB tags used in our projects. Vertical grey dashed line indicates the 0.8 threshold for data filtering. Note, for illustration purposes, the density axis of the generated plot was cut at 30 to achieve a better resolution for low densities.
