# Supplementary figures and images for: To cross or not to cross – thrushes at the German North Sea coast adapt flight and routing to wind conditions in autumn
Source: Mov Ecol. 2019 Oct 31;7:32. doi: 10.1186/s40462-019-0173-5 (PMC6824093; doi:10.1186/s40462-019-0173-5)

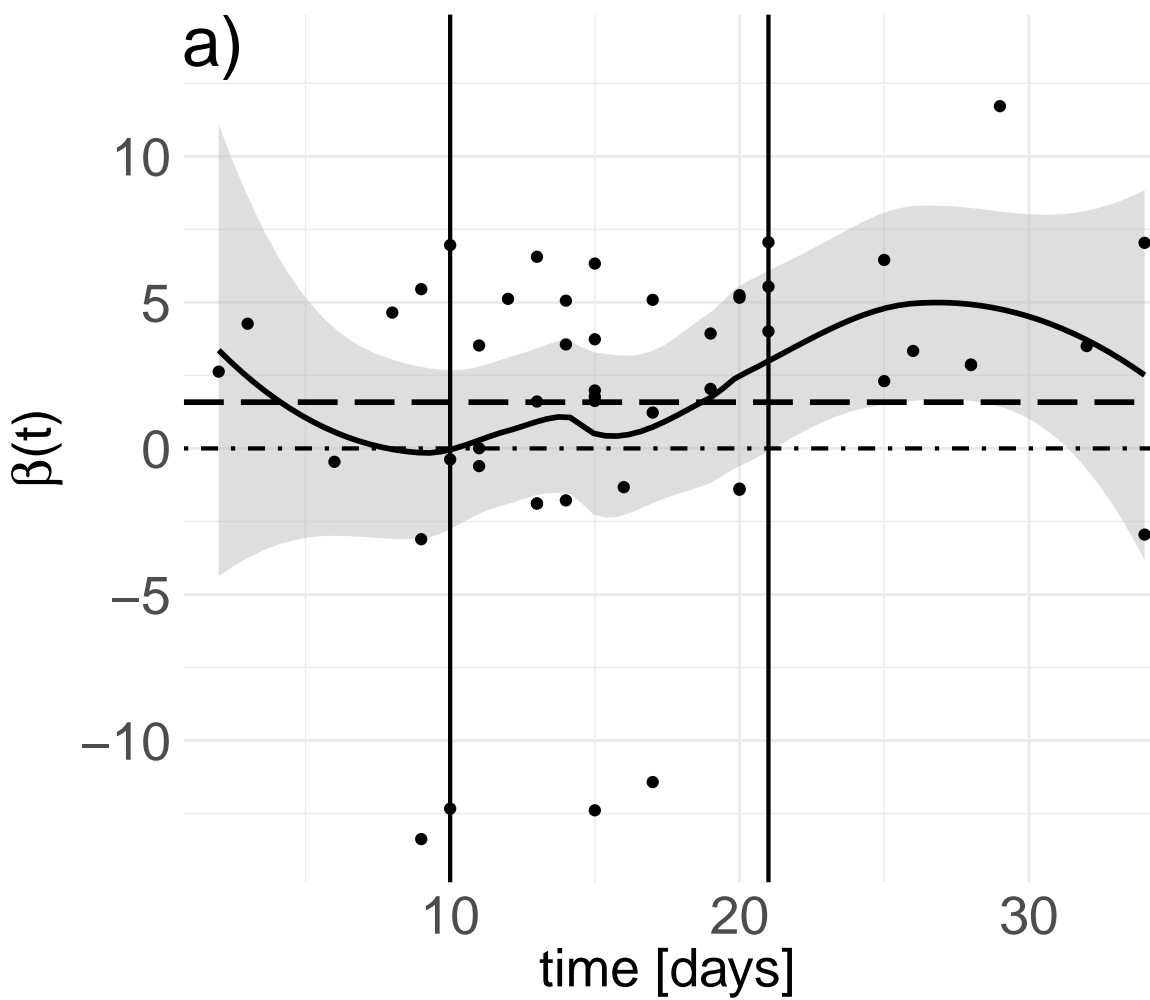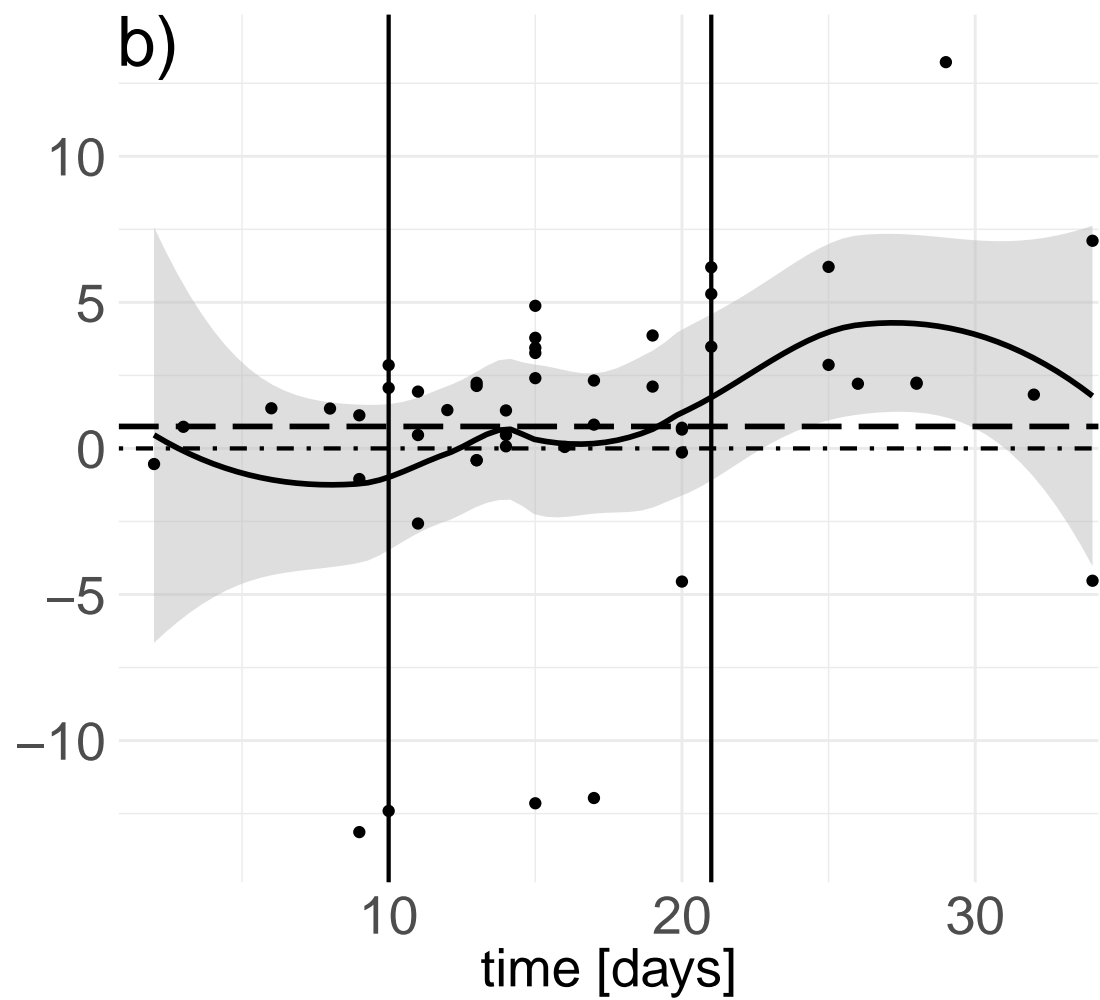

Supplement: Supplementary file 3 — Figure S2. Graphical test of the proportional hazards assumption of the β coefficients calculated in the Cox Proportional Hazards model, which showed a significant correlation over time. The graph displays Schoenfeld residuals (black dots) for β of species ‘redwing’ (a) and ‘song thrush’ (b) over time along with a smooth regression line and its 95% confidence intervals (grey shaded area). Zero as well as β calculated from the model are indicated as horizontal dot-dashed and dashed lines, respectively. Vertical lines indicate the post-hoc cut of the data at 10 and 21 days, respectively. (PDF 8 kb) [file 40462_2019_173_MOESM3_ESM.pdf]
